# Endothelial reactive oxygen-forming NADPH oxidase 5 is a possible player in diabetic aortic aneurysm but not atherosclerosis

**DOI:** 10.1038/s41598-022-15706-5

**Published:** 2022-07-07

**Authors:** Florence Ho, Anna M. D. Watson, Mahmoud H. Elbatreek, Pamela W. M. Kleikers, Waheed Khan, Karly C. Sourris, Aozhi Dai, Jay Jha, Harald H. H. W. Schmidt, Karin A. M. Jandeleit-Dahm

**Affiliations:** 1 Department of Diabetes, Central Clinical School, Monash University, 99 Commercial Road, Melbourne, VIC 3004 Australia; 2 Atherothrombosis and Vascular Biology Laboratory, Baker Heart and Diabetes Institute, 75 commercial Road, Melbourne, VIC 3004 Australia; 3 Department of Pharmacology and Toxicology, Faculty of Pharmacy, Zagazig University, Zagazig, 44519 Egypt; 4 Department of Pharmacology and Personalised Medicine, MeHNS, Faculty of Health, Medicine & Life Science, Maastricht University, Universiteitssingel 40, 6229 ER Maastricht, The Netherlands; 5 Institute for Clinical Diabetology, German Diabetes Centre, Leibniz Centre for Diabetes Research at Heinrich Heine University Düsseldorf, Auf’m Hennekamp 65, 40225 Düsseldorf, Germany

**Keywords:** Atherosclerosis, Aneurysm

## Abstract

Atherosclerosis and its complications are major causes of cardiovascular morbidity and death. Apart from risk factors such as hypercholesterolemia and inflammation, the causal molecular mechanisms are unknown. One proposed causal mechanism involves elevated levels of reactive oxygen species (ROS). Indeed, early expression of the ROS forming NADPH oxidase type 5 (*Nox5*) in vascular endothelial cells correlates with atherosclerosis and aortic aneurysm. Here we test the pro-atherogenic *Nox5* hypothesis using mouse models. Because *Nox5* is missing from the mouse genome, a knock-in mouse model expressing human *Nox5* in its physiological location of endothelial cells (eNOX5^ki/ki^) was tested as a possible new humanised mouse atherosclerosis model. However, whether just on a high cholesterol diet or by crossing in aortic atherosclerosis-prone *ApoE*^−/−^ mice with and without induction of diabetes, Nox5 neither induced on its own nor aggravated aortic atherosclerosis. Surprisingly, however, diabetic ApoE^−/−^ x eNOX5^ki/ki^ mice developed aortic aneurysms more than twice as often correlating with lower vascular collagens, as assessed by trichrome staining, without changes in inflammatory gene expression, suggesting that endothelial Nox5 directly affects extracellular matrix remodelling associated with aneurysm formation in diabetes. Thus *Nox5*-derived reactive oxygen species are not a new independent mechanism of atherosclerosis but may enhance the frequency of abdominal aortic aneurysms in the context of diabetes. Together with similar clinical findings, our preclinical target validation opens up a first-in-class mechanism-based approach to treat or even prevent abdominal aortic aneurysms.

## Introduction

Atherosclerosis with and without diabetes are a major cause of cardiovascular morbidity and death. Consequences include ischemic stroke, myocardial infarction, aortic aneurysm, and death. Despite the progress made in prevention and treatment of atherosclerosis, this disease and its consequences have grown globally and are the primary cause of deaths around the world^1,2^. Apart from elevated LDL cholesterol and a proinflammatory state, the causal pathomechanisms leading to atherosclerosis and allowing a curative therapy are not fully understood^3,4^.

One mechanism that has been suggested for decades to be causal in atherogenesis is dysregulated reactive oxygen species (ROS) formation^5–7^. ROS-scavenging antioxidants are, however, not effective, or beneficial overall, possibly due to also many protective effects of ROS and antioxidants interfering at the same time with protective and disease-triggering ROS^8^. Instead, an alternative approach that has been put forward is to identify and inhibit the disease-triggering enzymatic sources of ROS and leave beneficial enzymatic sources of ROS untouched^9^.

The only known dedicated enzymatic source of ROS is NADPH oxidase with five isozymes (Nox1-5). Nox1, at least preclinically plays only a minor role in both comorbidities^10–12^; knocking out Nox2 even increases infections and mortality in diabetes and is thus not a viable target^10^; Nox3 seems to be involved in pulmonary hypertension^13^; Nox4 is, surprisingly, rather atheroprotective^14,15^ and downregulated in abdominal aortic aneurysm^6^; Nox5 appear to stand out in atherosclerosis, at least by the clinical correlation of its expression levels in endothelial cells, both in early stages of coronary artery disease and aortic aneurysm^6,7,16^. However, the functional validation of this possible role of NOX5 in atherosclerosis is missing, possibly due to the fact that the *Nox5* gene is absent from the mouse and rat genome^16^. To test this Nox5 hypothesis of atherosclerosis, we investigated humanized *Nox5* knock-in (KI) mice expressing Nox5 in endothelial cells (eNOX5^ki/ki^)^17^, i.e., the physiological location in humans^18^.

## Results

### eNOX5 does not induce atherosclerosis per se

We first tested whether the knock-in of the *Nox5* gene per se can induce atherosclerosis in aged mice exposed to a high cholesterol Paigen diet, which is atherogenic in other aged mouse models^19^. After 28 weeks of diet, eNOX5^ki/ki^ mice had lower body weights compared to WT mice, whilst kidney, liver, and heart weights (Table 1) and blood glucose, serum triglycerides and cholesterol levels were similar (Table 1).

**Table 1:** Metabolic parameters in aged WT and eNOX5^ki/ki^ mice.

	Aged WT	Aged eNOX5^ki/ki^
Body weight, g	40.5 ± 1.9 (19)	35.8 ± 1.1 (16)*
Blood glucose, mmol/L	5.0 ± 0.2 (19)	4.6 ± 0.2 (17)
Cholesterol, mmol/L	1.4 ± 0.1 (19)	1.1 ± 0.1 (17)
Triglycerides, mmol/L	0.13 ± 0.02 (19)	0.15 ± 0.06 (17)
Kidney weight, g	0.51 ± 0.03 (19)	0.46 ± 0.02 (17)
Heart weight, g	0.2 ± 0.005 (19)	0.2 ± 0.008 (16)
Liver weight, g	2.4 ± 0.2 (18)	1.9 ± 0.1 (17)

Data are shown as mean ± SEM (n). *P* < 0.05 * compared with aged WT.

**Figure 1:**
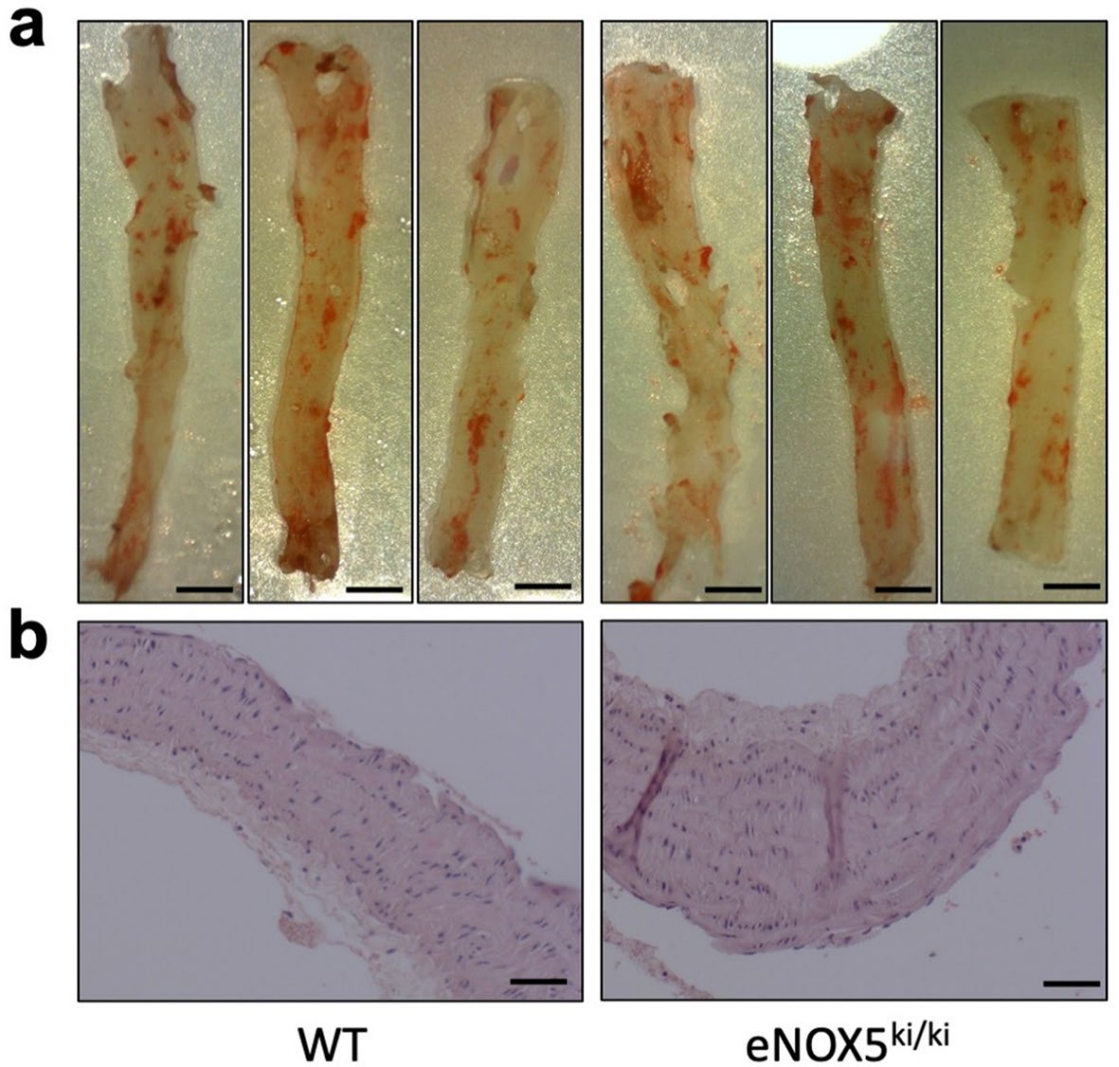
No plaques or fat infiltration in the aorta of the aged eNOX5^ki/ki^ mice. Representative images of aorta of aged WT and eNOX5^ki/ki^ mice stained with Sudan stain (**a**) and aortic arch stained with H/E stain (**b**). a. Absence of plaques or fat infiltration in the aorta of the aged WT and eNOX5^ki/ki^. b. No fatty streaks or plaque formation were seen in the aortic arch of the aged WT and eNOX5^ki/ki^. Plates are representative of n = 4–5 experiments. Bar indicates 2 mm (**a**) and 50 µm (**b**).

We then measured the atherosclerotic plaque area in the whole aorta of both WT and eNOX5^ki/ki^ mice by en-face analysis. However, there was no plaque formation in both groups (Fig. 1a). We then considered that Nox5 might be involved only in the early stages of atherosclerosis development, i.e., fatty streak formation at the aortic arch^20^. To test this, we stained the aortic arch with an Haemotoxylin and Eosin stain providing a comprehensive picture of the microanatomy. However, as before we did not detect any fatty streaks in WT and eNOX5^ki/ki^ mice (Fig. 1b). Also, aneurysms were not observed (not shown). These data indicated that endothelial expression of Nox5 per se is insufficient to develop fatty streaks, advanced atherosclerotic lesions, or aneurysms, even if mice are aged and exposed to an otherwise atherogenic diet. Thus, we next decided to retest the Nox5 hypothesis of atherosclerosis under conditions where atherosclerosis is observed and determine whether *Nox5* KI aggravates this phenotype in the atherosclerosis prone apolipoprotein E (ApoE^−/−^) mouse in both normal and diabetic conditions.

### eNOX5 does not aggravate atherosclerosis in ApoE^−/−^ mice with and without diabetes

Mice deficient in apo E (ApoE^−/−^ mice) spontaneously develop atherosclerosis^21,22^. Therefore, eNOX5^ki/ki^ mice were crossed with ApoE^−/−^ mice to test the role of Nox5 under aggravated conditions. Moreover, given the role of Nox5 in diabetic complications^23,24^ and because atherosclerosis is accelerated in diabetes, we further induced diabetes in some of the eNOX5^ki/ki^ x ApoE^−/−^ mice by the injection of low-dose streptozotocin (STZ). After 20 weeks, all diabetic mice (both WT and eNOX5^ki/ki^) had lower body weights (Table 2), elevated glucose, HbA1c, total cholesterol and LDL levels, and similar blood pressure compared to their nondiabetic controls. There were no significant differences in all of the above-mentioned parameters between WT and eNOX5^ki/ki^ mice with or without diabetes, however, diabetic eNOX5^ki/ki^ mice had slightly higher body weights. These data show that endothelial expression of Nox5 has no effect on metabolic parameters and blood pressure in ApoE^-/-^ mice with/without diabetes (Table 2).

**Table 2:** Baseline data including metabolic parameters for animals at 20 weeks of study.

	Control ApoE^−/−^xWT	Control ApoE^−/−^x eNOX5^ki/ki^	Diabetic ApoE^−/−^ x WT	Diabetic ApoE^−/−^x eNOX5^ki/ki^	*p*-value: Effect of diabetes	*p*-value: Effect of NOX5
Body weights (g)	30.6 ± 1.0	31.6 ± 1.1	25.1 ± 0.3 #†	28.8 ± 0.5 ‡	**< 0.0001**	**0.0110**
24 h urine output (mL)	1.0 ± 0.1	0.9 ± 0.2	16.0 ± 1.6 #†	16.8 ± 2.7 #†	**< 0.0001**	0.8490
HbA1c (mmol/mol, %)	4.6 ± 0.2	4.4 ± 0.1	10.1 ± 0.6 #†	9.6 ± 0.3 #†	**< 0.0001**	0.3560
Plasma glucose (mmol/L)	9.3 ± 0.8	9.8 ± 0.7	21.6 ± 2.2 #†	24.1 ± 2.8 #†	**< 0.0001**	0.3957
Cholesterol (mmol/L)	14.1 ± 0.5	11.5 ± 0.3	23.7 ± 4.6 #†	22.5 ± 1.7 #†	**< 0.0001**	0.3516
Triglycerides (mmol/L)	1.3 ± 0.1	0.8 ± 0.1	1.6 ± 0.3	1.3 ± 0.2 ‡	0.0947	**0.0448**
Plasma HDL (mmol/L)	1.6 ± 0.0	1.5 ± 0.1	1.0 ± 0.2 #†	0.8 ± 0.2 #†	**< 0.0001**	0.3431
Plasma LDL (mmol/L)	9.9 ± 4.6	12.0 ± 1.6	21.1 ± 0.5 #†	22.1 ± 0.2 #†	**< 0.0001**	0.4616
Tibia lengths (mm)	17.2 ± 0.2	17.6 ± 0.1	17.5 ± 0.1	17.4 ± 0.1	0.8589	0.4300
Systolic blood pressure (mmHg)	105 ± 27	105 ± 27	94 ± 30	107 ± 36	0.3421	0.1783

Data shown as mean ± SEM. Two-way ANOVA statistics shown in columns. Post-hoc multiple comparisons test (Tukey’s): # versus Control ApoE^−/−^ x WT group; † versus Control ApoE^−/−^ x eNOX5^ki/ki^ group; ‡ versus Diabetic ApoE^−/−^ x WT group. n = 6–10/group for body weight, 24 h urine output and HbA1c values; n = 7–9/group for plasma values; n = 8–18/group for tibia lengths; n = 9–15 for blood pressure measurements. The first *p* value “the effect of diabetes” is for the comparison between non-diabetic WT mice and diabetic WT mice. The second *p* value “the effect of NOX5” is for the comparison between diabetic WT mice and diabetic NOX5 mice. ApoE^−/−^ = Apolipoprotein E knockout; HDL = high density lipoprotein; LDL = low density lipoprotein; NOX = NADPH oxidase. Significant values are bold

**Figure 2:**
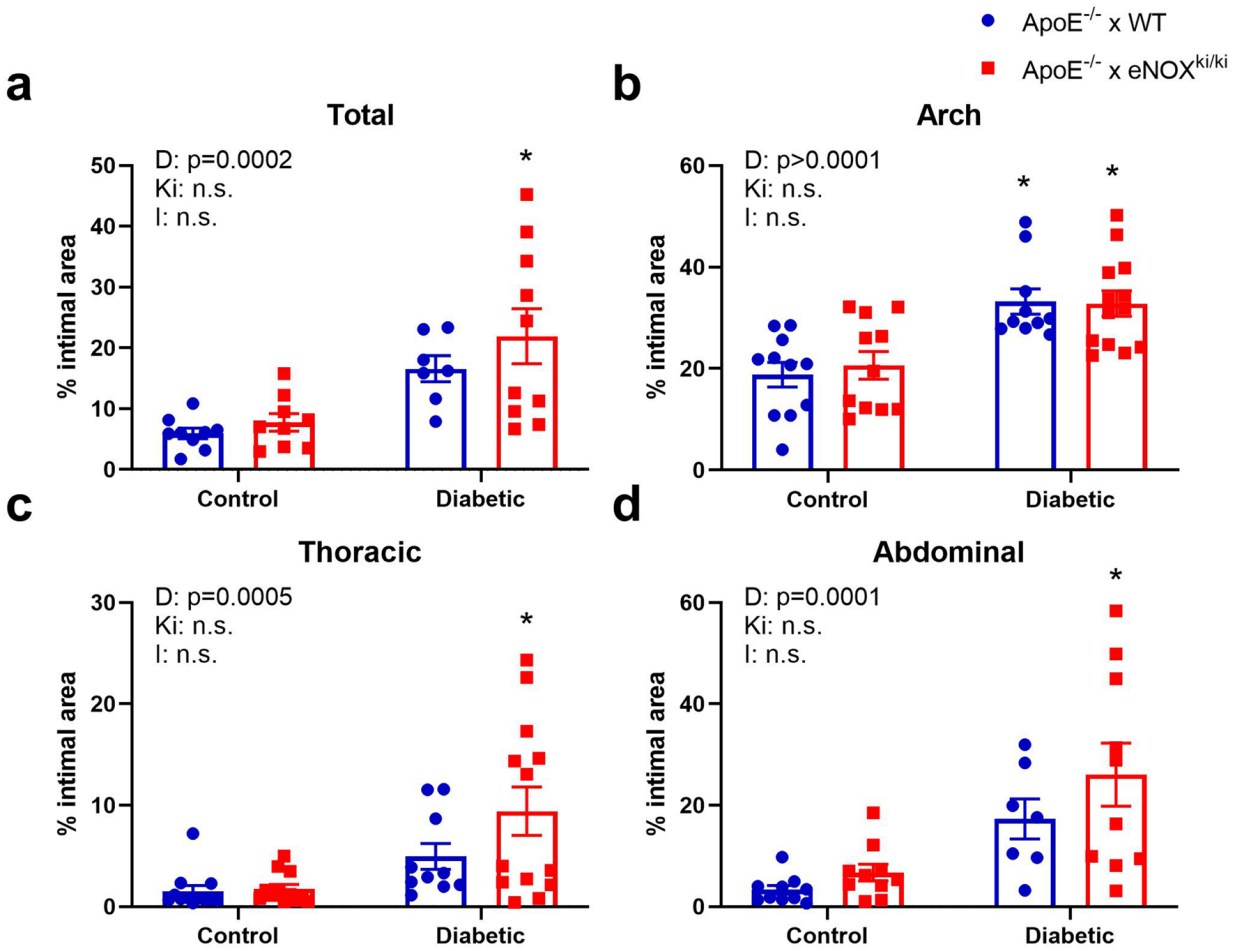
Endothelial NOX5 does not potentiate atherosclerosis in non-diabetic and diabetic ApoE^−/−^ mice. Atherosclerotic plaque area was measured at 20 weeks after induction of diabetes in total aorta (**a**), aortic arch (**b**), thoracic aorta (**c**) and abdominal aorta (**d**). Animals with diabetes showed significantly greater levels of atherosclerosis however animals expressing endothelial Nox5 (red bars) did not have significantly different levels of intimal plaque area compared to ApoE^−/−^ WT (blue bars). Data shown as mean ± SEM of n = 7–10 animals/group. 2-way ANOVA: D- relative to diabetic status; Ki- relative to genotype (Nox5); I- interaction. * Tukey t-test versus relevant non-diabetic control *P* < 0.05. Representative images of *en face* mouse aorta from ApoE^−/−^ mice stained with sudan IV are shown in Supplementary Fig. S5 online.

When assessing the atherosclerotic plaque area in ApoE^−/−^ x WT versus ApoE^−/−^ x eNOX5^ki/ki^ mice with and without 20 weeks of diabetes, all diabetic mice showed a significant increase in atherosclerotic plaque area in total, arch, thoracic and abdominal aorta compared to non-diabetic mice, yet there was no difference between diabetic ApoE^−/−^ x WT versus diabetic ApoE^−/−^ x eNOX5^ki/ki^ mice in all areas (Fig. 2).

### eNOX5 increases aneurysms in diabetic ApoE^−/−^ mice

**Figure 3:**
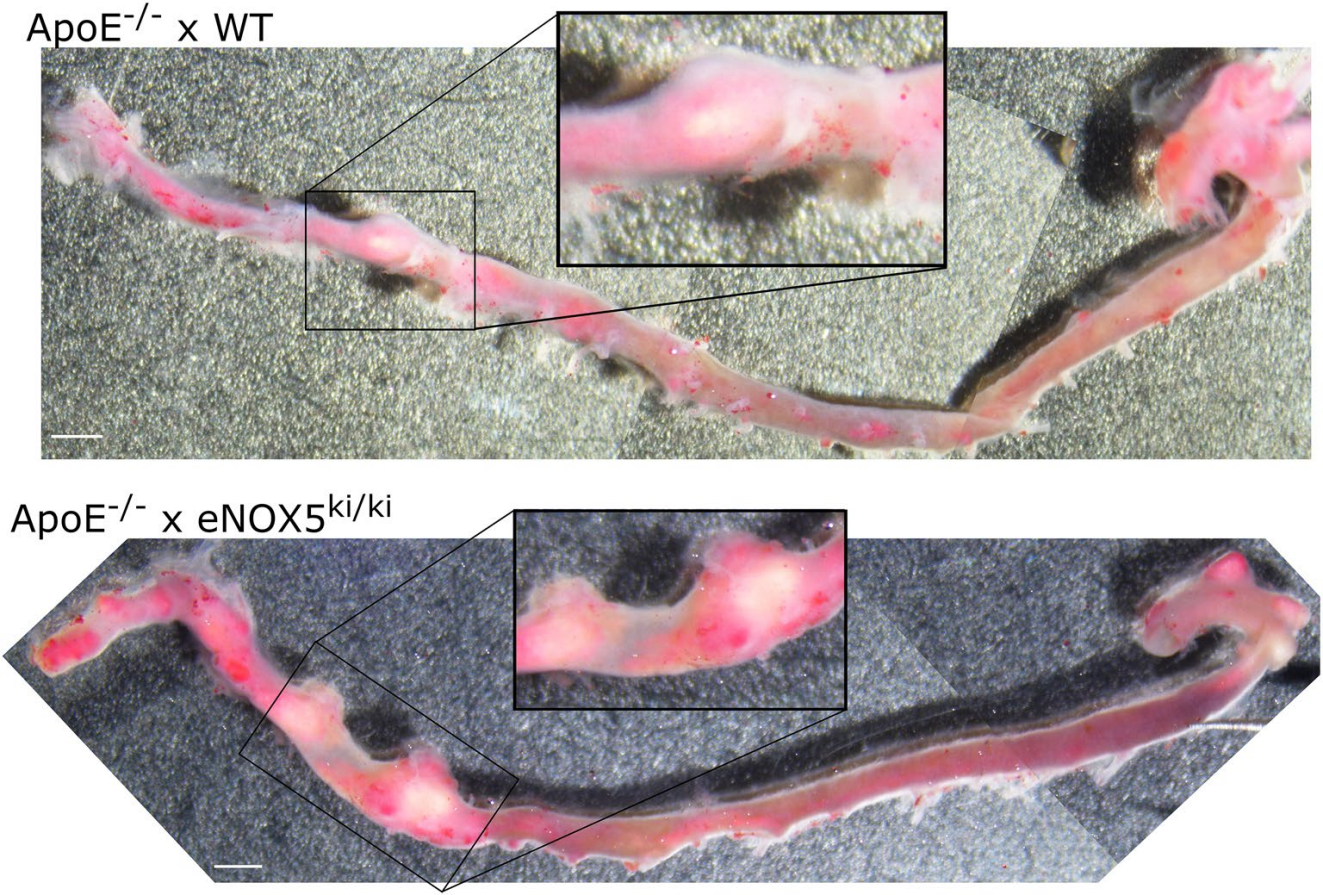
Whole mount examples of sudan IV stained aortas of diabetic ApoE^−/−^ mice with and without endothelial NOX5 expression. Bars (white, at bottom left) indicate 1 mm.

Finally, based on the clinical observations of Nox5 levels correlating with aortic aneurysms^6,7^, we investigated whether Nox5 induces aortic aneurysm under atherosclerotic conditions with and without diabetes. Indeed, diabetic ApoE^−/−^ x eNOX5^ki/ki^ mice formed more frequently aneurysms than ApoE^−/−^ WT mice (Table 3, Fig. 3). According to the Daugherty classification, the aneurysms were type II^25^. The combination of Nox5 expression and diabetes was associated with a significant increase in aneurysm incidence compared to non-diabetic Nox5 expressing mice (Table 3, *p* < 0.01).

**Table 3:** Aneurysm formation in diabetic ApoE^−/−^ x NOX5^ki/ki^ animals and their respective WT controls at 20 weeks of study.

	Animals with aneurysms		Average expansion
	Absolute n with/without	%	%
Non-diabetic ApoE^−/−^ x WT	1/21	4.8%	178%
Non-diabetic ApoE^−/−^ x eNOX5^ki/ki^	0/32	0.0%	n/a
Diabetic ApoE^−/−^ x WT	3/24	12.5%	144 ± 3%
Diabetic ApoE^−/−^ x eNOX5^ki/ki^	9/32	28.1%*	155 ± 8%

Data are expressed as the number of animals with aneurysms present, compared to the total number of animals assessed for aneurysm formation. Average % expansion as compared to normal vessel width measured above and below the aneurysm (where normal vessel = 100%); n for average expansion: nondiabetic ApoE^−/−^ x WT = 1, Diabetic ApoE^−/−^ x WT = 3, Diabetic ApoE^−/−^ x eNOX5^ki/ki^ = 8.

Overall significant in a non-parametric multiple comparison (Kruskal Wallis test).

*p < 0.01 Diabetic ApoE^−/−^ x eNOX5^ki/ki^ versus non-diabetic ApoE^−/−^ x eNOX5^ki/ki^.

To explore the possible downstream mechanisms of this Nox5-dependent effect, we examined gene expression of inflammatory, oxidative/antioxidant and fibrotic markers in the aorta (Table 4). Diabetes induced an increase in the expression of monocyte chemoattractant protein-1 (MCP-1), the macrophage marker F4/80, nitrotyrosine, Nox2, heme oxygenase-1 (HO-1) and platelet derived growth factor (PDGF) (Table 4). Notably, there was a trend towards reduced Nox2 expression in the diabetic eNOX5^ki/ki^ mice compared to WT mice, yet there was no significant difference between both groups.

**Table 4:** RT-PCR gene expression data for ApoE^−/−^ x NOX5^ki/ki^ animals and their respective WT controls at 10 weeks of study.

Gene of interest	Control ApoE^−/−^x WT	Control ApoE^−/−^x eNOX5^ki/ki^	Diabetic ApoE^−/−^ x WT	Diabetic ApoE^−/−^ x eNOX5^ki/ki^	*p*-value: Effect of diabetes	*p*-value: Effect of NOX5
**Inflammatory markers**						
MCP-1	1.00 ± 0.41	0.94 ± 0.96	2.49 ± 0.45	5.21 ± 1.71 #†	**0.0031**	0.1458
VCAM-1	1.00 ± 0.41	0.43 ± 0.11	0.96 ± 0.27	1.99 ± 0.67	0.0816	0.5834
F4/80	1.00 ± 0.29	0.92 ± 0.18	4.95 ± 1.84 #†	2.22 ± 0.54	**0.0062**	0.1200
NFkB (transcription factor p65, RelA)	1.00 ± 0.25	1.06 ± 0.16	1.00 ± 0.17	0.90 ± 0.33	0.7602	0.9980
**NOX isoforms and antioxidants**						
NOX2	1.00 ± 0.22	1.36 ± 0.21	9.71 ± 3.68 #†	3.81 ± 0.70	**0.0020**	0.0977
NOX4	1.00 ± 0.30	0.65 ± 0.12	0.58 ± 0.13	0.71 ± 0.35	0.4581	0.6650
HO1	1.00 ± 0.18	1.68 ± 0.22	3.52 ± 0.87 #	3.20 ± 0.70 #	**0.0005**	0.7158
GPx1	1.00 ± 0.12	1.03 ± 0.10	1.59 ± 0.36	0.95 ± 0.14	0.2097	0.1398
NRF2	1.00 ± 0.13	1.40 ± 0.19	1.79 ± 0.99	1.76 ± 0.78	0.2609	0.7159
**Fibrotic and remodelling markers**						
Collagen III	1.00 ± 0.26	1.15 ± 0.17	1.83 ± 0.50	1.09 ± 0.32	0.2300	0.3549
Collagen IV	1.00 ± 0.24	0.71 ± 0.05	1.10 ± 0.29	0.57 ± 0.18	0.9192	0.0692
Fibronectin	1.00 ± 0.23	0.92 ± 0.14	2.91 ± 0.96 †	0.71 ± 0.13 ‡	0.1074	**0.0347**
CTGF	1.00 ± 0.18	1.04 ± 0.17	0.84 ± 0.33	0.75 ± 0.38	0.3869	0.9190
MMP2	1.00 ± 0.27	1.18 ± 0.32	2.10 ± 0.65	1.38 ± 0.25	0.1202	0.5085
MMP9	1.00 ± 0.38	0.91 ± 0.11	0.61 ± 0.14	1.09 ± 0.58	0.7800	0.6144
PKC alpha	1.00 ± 0.13	1.07 ± 0.24	1.73 ± 0.35	1.14 ± 0.35	0.1539	0.3420
PDGF	1.00 ± 0.25	1.68 ± 0.21	2.29 ± 0.88	3.61 ± 1.26	**0.0379**	0.1818

Data shown as mean ± SEM, with all values expressed relative to non-diabetic NOX5 negative animals. Two-way ANOVA statistics shown in columns, in bold if *p* < 0.05. Post-hoc multiple comparisons test (Tukey’s): # versus Control ApoE^−/−^ x WT group; † versus Control ApoE^−/−^ x eNOX5^ki/ki^ group; ‡ versus Diabetic ApoE^−/−^ x WT group. n = 6–9/group (except n = 5 for the diabetic NOX5 negative groups; for NRF2, n = 5 for the diabetic eNOX5^ki/ki^ group for CTGF, n = 5 for diabetic NOX5 negative group for MMP9). The first *p* value “the effect of diabetes” is for the comparison between non-diabetic WT mice and diabetic WT mice. The second *p* value “the effect of NOX5” is for the comparison between diabetic WT mice and diabetic NOX5 mice.

Further, immunohistochemistry showed higher VCAM-1, nitrotyrosine and MCP-1 in diabetic mice compared to nondiabetic mice (See Supplementary Fig. 1 online and Supplementary Fig. 2 online). Yet, there was no difference between WT versus eNOX5^ki/ki^ mice with/without diabetes in all markers (Table 4, See Supplementary Fig. 1 online).

**Figure 4:**
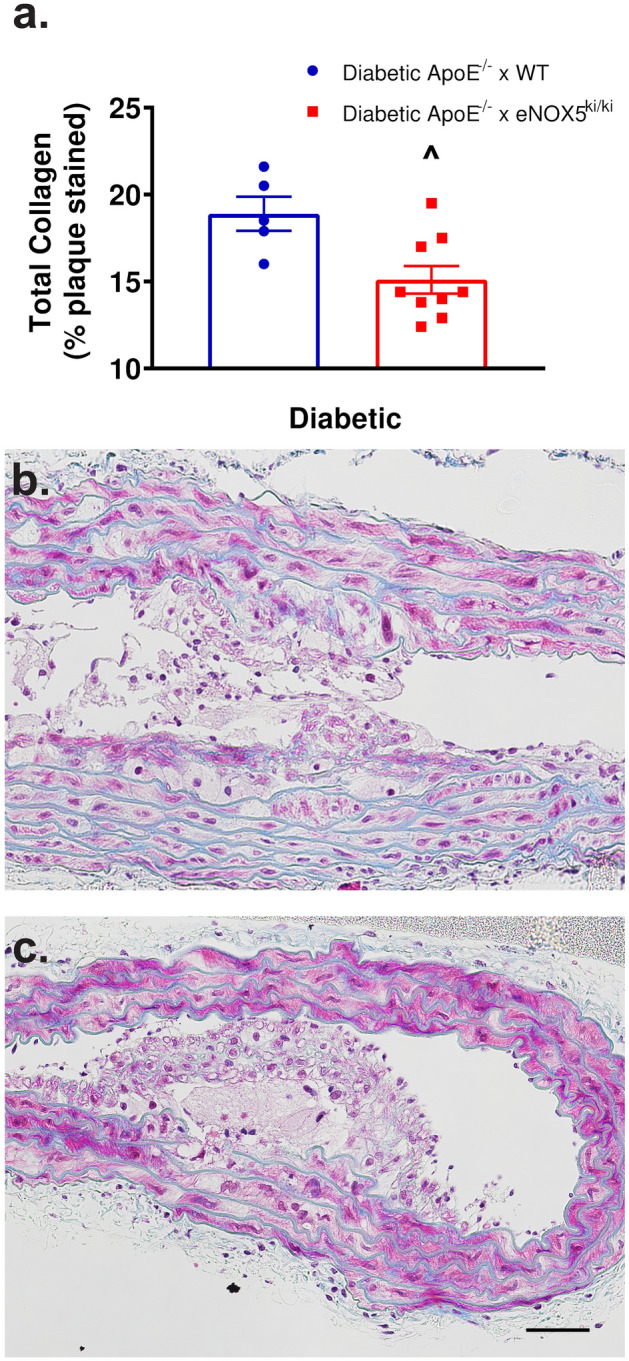
Endothelial Nox5 expression results in lower vascular collagen accumulation in diabetic eNOX5^ki/ki^ApoE^−/−^ mice (**a**). Quantification of trichrome staining in the atherosclerotic plaque and underlying vessel wall of diabetic ApoE^−/−^ mice with (**b**) and without (**c**) eNOX5 expression. Assessment of the amount of blue collagen accumulation shows that diabetic animals expressing eNOX5 had significantly less collagen. Data shown as mean ± SEM of n = 6–9 animals/group. ^ Student’s t-test between diabetic groups *p* = 0.012. 50 μm scale bar.

However, diabetic ApoE^−/−^ x eNOX5^ki/ki^ mice showed lower fibronectin gene expression compared to diabetic ApoE^−/−^ x WT mice (Table 4). This, however, did not translate to the protein level, where fibronectin protein expression was not significantly different between diabetic eNOX5^ki/ki^ ApoE^−/−^ and WT ApoE^−/−^ mice (See Supplementary Fig. 3 online), whereas collagen accumulation in the plaques and underlying vascular wall was significantly reduced in diabetic ApoE^−/−^ x eNOX5^ki/ki^ compared to diabetic ApoE^−/−^ WT mice. This suggests that in diabetic mice, eNOX5 induces aneurysm not via an inflammatory mechanism but altered extracellular matrix remodelling (Fig. 4).

### Discussion

In search for a causal mechanism in atherosclerosis, the superoxide-forming Nox5 was such a highly promising candidate. It is overexpressed in the endothelium in early atherosclerotic lesions, in vascular smooth muscle cells in the advanced coronary lesions in patients with coronary artery disease^16^, and in human abdominal aortic aneurysm^6,26^. The calcium-dependent activation of Nox5 and ROS generation in endothelial cells has been addressed by previous studies^27,28^. Besides, in our previous study^18^, we investigated the eNox5^ki/ki^ mouse model in acute model of ischemic stroke which is inherently characterized by excitotoxicity and increased calcium levels.

However, despite its attractiveness and considerable circumstantial clinical evidence, our data argue against a previously unrecognized direct causal or aggravating role of Nox5 in diet-related or ApoE^−/−^ induced atherosclerosis. Thus, the eNOX5^ki/ki^ mouse cannot be a humanized mechanism-based model for atherosclerosis as we initially expected.

This observation is in line with a recent observation published, whilst the present manuscript was in preparation, on an elegant knock-out study in rabbits. Unlike mice and rats, rabbits do express NOX5 physiologically. Here, deleting the Nox5 gene was not only neutral but even aggravated atherosclerosis^29^. Whilst our model has the limitation of only mimicking physiological human expression of Nox5 in endothelial cells, the Nox5 KO rabbit model is based entirely on endogenous expression levels and localization. Thus, our and the rabbit data clearly argue against a general pro-atherosclerotic role of Nox5, despite the earlier observed expressional correlation^16^. These data are similar to those by us and others studying Nox4, which is also neutral to protective^14,15^. We cannot rule out a role for another Nox isoform, e.g., Nox1 or Nox2, but the possibility exists that ROS formation in atherosclerosis is not causal but downstream or an epiphenomenon.

The rather unexpected finding of this study was the clear causal role of endothelial Nox5 in type II^25^ abdominal aortic aneurysms under diabetic conditions. This was reminiscent of the role of Nox5 in other atherosclerosis-associated comorbidities, i.e., diabetic kidney disease^24,30^, hypertension^17^, stroke^18^ and myocardial infarction^31^. Indeed, diabetes is a negative risk factor of aortic aneurysms^32^ and anti-diabetic treatment regimens afford protection^33^ as a function of changes in the vascular extracellular matrix and a more fibrotic phenotype^32,34^. However, other Nox isoforms cannot be excluded as in human aortic aneurysm, mRNA levels for Nox2 and Nox5 are significantly increased, and expression of potentially protective Nox4 mRNA decreased^6^. Moreover, deletion of Nox1 in mice attenuates angiotensin II-induced aortic aneurysm formation^12,35^, which may however, differ from the diabetes-dependent mechanism. Obviously, in our study, Nox2 levels in eNOX5^ki/ki^ mice were lower than the diabetic WT mice, yet the difference was not significant. Although unlikely, we cannot exclude that changes in Nox2 expression also play a role in the NOX5-induced aortic aneurysms in diabetes, however, further studies, e.g., focusing on NOX2 protein levels, are needed to support this conclusion. As a limitation of our study, we have not measured the expression of other NOX isoforms including Nox1, therefore, its downstream contribution to the primary effect of NOX5 cannot be ruled out. Furthermore, the pro-inflammatory marker MCP-1 was significantly increased in diabetic mice and was almost double in diabetic ApoE^−/−^ x eNOX5^ki/ki^ mice compared to diabetic ApoE^−/−^x WT mice, however this increase was not significant.

Downstream of endothelial Nox5, reducing collagen formation may be involved. This would, however, be atypical for Nox5 as, for example, in diabetic kidney disease^24^ and human hepatic stellate cells^24,30^, Nox5 is associated with rather increased extracellular matrix proteins^24,30^ and fibrosis^36^, respectively. Thus, our study has limitations as the downstream mechanisms of Nox5 aggravating diabetic aneurysm remain unclear. Indeed, insoluble form of fibronectin is expressed in endothelial cells^37^ and its polymerization into the extracellular matrix is required for the deposition of collagen^38,39^. This remarkable finding agrees with our results in diabetic mice, where the diabetes-induced overexpression of fibronectin was prevented by knocking in Nox5. Thus, investigation of this effect in vitro, in endothelial cells, is warranted in future studies. Moreover, fibronectin is upregulated in many cancers including non-small cell lung carcinoma (NSCLC)^40,41^. This suggests that Nox5 may protect against cancer by reducing fibronectin, and that tumor tendency may be lower in eNOX5^ki/ki^ mice. Therefore, future studies of cancers, in particular NSCLC, in Nox5 KI mice expressing Nox5 in endothelial cells and other cell types is warranted.

Despite the effect of Nox5 on fibrotic markers, it had no effect on *F4/80* and *NOX2* expression between diabetic WT and diabetic eNOX5^ki/ki^ mice. However, there was a tendency for lower expression of both genes in eNOX5^ki/ki^ mice, yet not significant. Thus, there might be a dysfunction of macrophage attraction due to overexpression of Nox5, which is also expressed in macrophages of eNOX5^ki/ki^ mice according to our previous publications^18^, although there was a tendency for an increased *MCP-1* expression in the diabetic eNOX5^ki/ki^ group.

As an outlook, it would be of interest to examine the role of Nox5 in other models and types^25^ of abdominal aortic aneurysm, e.g., ApoE^−/−^ mice infused with angiotensin II^25,42^. Rather than the down-stream mechanistic consideration, it will be of interest to test Nox5 specific inhibitors for their therapeutic potential once they become available^43,44^.

### Conclusion

The relevance and precise enzymatic source of ROS in atherosclerosis remains unclear. Unlike previous correlations had suggested, endothelial Nox5 is not a relevant source. Instead, Nox5 may be an aggravating or even causal factor in the human diabetic complication of aortic aneurysms, which have a pre-/in-hospital mortality of 40–80%. Given the fact that Nox inhibitors are beginning to be clinically tested our findings may provide a first step towards a mechanism-based, isoform-specific therapy or even prevention of aortic aneurysms.

## Methods

### Animals

Humanized endothelial Nox5 Knock-in (KI) mice were generated and validated as previously described^18^. Briefly, the model was developed using the hypoxanthine phospho-ribosyl-transferase (Hprt) targeted transgenic approach under the control of the Tie2 promoter. Therefore, Nox5 is expressed endogenously mainly in endothelial cells and white blood cells (eNOX5^ki/ki^) mimicking the physiological expression pattern and levels as in humans. This technology is superior to transgenic animals, e.g., as reported for NOX2 overexpressing mice, where the target gene is randomly inserted and expressed globally in supraphysiological levels^45–47^. The mice had a mixed genetic background (129/Sv and C57Bl6). Expression of Nox5 in the KI mice tissues was validated by qPCR and compared to Wild Type (WT) mice^18^. Both WT and eNOX5^ki/ki^ mice (56–64 weeks old) (total n = 42) were fed a high cholesterol (Paigen) diet (without cholate) for 28 weeks to induce atherosclerosis. On the day of sacrifice, blood glucose was measured by using a glucometer (Contour XT, Ascensia, Switzerland), mice were then anesthetized with 3–4% isoflurane. The abdominal cavity was opened, and blood was withdrawn via a heart puncture. Then, the mouse was flushed with 20 ml nitroprusside and organs were taken out and weighed. The blood collected from the mice was allowed to clot for 30 min to 1 h. After 10 min centrifugation at 10.000 rpm at 4 °C, the supernatant (serum) was pipetted and aliquoted in 100μL portions to be stored at − 20 °C. Cholesterol levels were measured by Cholesterol FS 10` kit (DiaSys–Diagnostic Systems GmbH, Holzheim, Germany). Triglyceride levels were measured by Triglyceride FS 5` Ecoline kit (DiaSys–Diagnostic Systems GmbH, Holzheim, Germany). All aged mice experimental protocols were approved by the Animal Ethics Committee of the Faculty of Health, Medicine and Life Sciences, Maastricht University, Netherlands. All experiments were performed in accordance with the ARRIVE guidelines (“Animal Research: Reporting of In Vivo Experiments”; https://arriveguidelines.org/).

eNOX5^ki/ki^ mice were also bred against ApoE^−/−^/C57Bl6 mice with all mice genotyped. SNP analysis showed that mice were 91% similar to C57Bl6 (AGRF, St Lucia, QLD, Australia). Nox5 expression in endothelial cells was confirmed by immunofluorescence (See Supplementary Fig. 6 online). Mice were bred and housed at the Alfred Medical Research Education Precinct (AMREP) Animal Services animal house (Melbourne, VIC, Australia) under the approval of the AMREP ethics committee and studies were conducted according to Australian National Health and Medical Research Council guidelines in line with international standards. All mice had unrestricted access to water and feed, were maintained on a 12 h light–12 h dark cycle and were fed standard mouse chow (Barastoc, Ridley Corp., Melbourne, VIC, Australia) with water ad. lib. Animals were randomly allocated to remain non-diabetic (non-diabetic control) or be rendered diabetic: Non-diabetic ApoE^−/−^ x WT, non-diabetic ApoE^−/−^ x eNOX5^ki/ki^, diabetic ApoE^−/−^ x WT and diabetic ApoE^−/−^ x eNOX5^ki/ki^. Six-8-week-old WT (ApoE^−/−^) and ApoE^−/−^NOX5^ki/k^ mice were given streptozotocin (5 × 55 mg/kg daily i.p.; AdipoGen, Sapphire Bioscience, Redfern, NSW, Australia). Only diabetic mice with blood glucose > 15 mmol/L and HbA1c > 53 mmol/mol (7.0%; Cobas b 101, Roche Diagnostics, Mannheim, Germany) were included in the study. Systolic blood pressure was measured at 19 weeks after diabetes induction (tail-cuff; CODA, Kent Scientific, Torrington, CT, USA). At least 24 h after BP analysis, animals underwent 24 h metabolic caging. After 10 or 20 weeks of diabetes mice were given 100 mg/kg sodium pentobarbitone i.p (Lethobarb, Virbac Australia, Milperra, NSW, Australia), or gassed with carbon dioxide. Cardiac blood was collected and organs rapidly dissected. Plasma was separated and frozen for later lipid and glucose analysis (Beckman autoanalyzer, as described previously^48^). Aorta were stripped of adventitial fat under a dissecting microscope whilst being inspected for the presence of aneurysms and either fixed in 10% neutral buffered formalin for *en face* analysis before embedding in paraffin, or snap frozen in liquid nitrogen for RT-PCR.

### Atherosclerotic plaque area

Assessment of plaque area was undertaken using *en face* analysis, after staining with Sudan IV-Herxheimer’s solution (BDH, Poole UK) as previously described^49^.

### Hematoxylin and Eosin stain

4um thick sections of paraffin embedded aortic arches and valvular heart region, were cut and stained with Hematoxylin and Eosin to detect fatty streaks and/or plaques in the aortic arch and valvular heart region.

### Quantitative RT-PCR

Total RNA was extracted after homogenising whole aorta (Polytron PT-MR2100; Kinematica, Littau/Lucerne, Switzerland) in TRIzol reagent (Invitrogen Australia, Mt Waverley, Vic, Australia) as previously described^38^. Gene expression was expressed relative to 18S and analyzed by comparative CT with as previously described^38^. See supplementary Table 1 for probe and primer sequences.

### Immunohistochemical and trichrome staining

Immunohistochemical and trichrome staining was performed on 4 μm paraffin embedded sections as described previously^45,47^. Rabbit anti-fibronectin (1:1000, Dako#A024502-2, ELITech Group, Braeside, VIC, Australia), rabbit anti-monocyte chemoattractant protein-1 (MCP-1; #ab7202, Abcam, Cambridge, MA, USA), rabbit anti-nitrotyrosine (1:200, Chemicon #ab411, Merck, Darmstadt, Germany), rabbit anti-vascular cell adhesion molecule-1 (VCAM-1; 1:50, #ab134047, Abcam). Photomicrographs and analysis for percentage collagen staining in the plaque and wall immediately adjacent to the plaque (exclusive of adventia) was conducted as described previously^38,40^ (Nikon Eclipse Ci microscope (Nikon, Tokyo, Japan) with a DS-Fi3 digital camera (Nikon, Tokyo, Japan) using Nikon NIS Elements software (Nikon); ver. 7.0, MediaCybernetics for percentage area of blue staining assessed in RGB). Note one data point represents the average of all sections present from one animal. Non-diabetic animals have significantly less plaque than diabetic mice; Fig. 4 represents the average collagen content of 2 or more plaques per animal.

### Statistical analysis

Data were analyzed for normality using the D'Agostino-Pearson test before being analyzed. T-test was used to compare between two groups and one or two-way ANOVA followed by Tukey’s multiple comparisons test to compare between more than two groups. *P* < 0.05 was considered significant. Results are expressed as mean ± SEM. Data were analyzed using Prism GraphPad software.

## Supplementary Information

Supplementary Information.

## Data Availability

All data needed to evaluate the conclusions in the paper are present in the paper or the Supplementary Materials.
