# Supplementary material for: Endothelial reactive oxygen-forming NADPH oxidase 5 is a possible player in diabetic aortic aneurysm but not atherosclerosis
Source: Sci Rep. 2022 Jul 7;12:11570. doi: 10.1038/s41598-022-15706-5 (PMC9262948; doi:10.1038/s41598-022-15706-5)
Supplement: Supplementary file 1 — Supplementary Information. [file 41598_2022_15706_MOESM1_ESM.pdf]

**Supplementary Information**

# Endothelial reactive oxygen-forming NADPH oxidase 5 is a possible player in diabetic aortic aneurysm but not atherosclerosis

Florence Ho[1,6], Anna Watson[1,2,6], Mahmoud H Elbatreek[3,4,6*], Pamela Kleikers[4], Waheed Khan[1], Karly C. Sourris[1], Aozhi Dai[1], Jay Jha[1], Harald H.H.W. Schmidt[4*], Karin Jandeleit-Dahm[1, 5*]

[1] Department of Diabetes, Monash University, Central Clinical School, 99 Commercial Road, Melbourne 3004, Victoria, Australia

[2] Atherothrombosis and Vascular Biology Laboratory, Baker Heart and Diabetes Institute, 75 commercial Road, Melbourne 3004, Victoria, Australia

[3] Department of Pharmacology and Toxicology, Faculty of Pharmacy, Zagazig University, 44519 Zagazig, Egypt.

[4] Department of Pharmacology & Personalised Medicine, MeHNS, Faculty of Health, Medicine & Life Science, Maastricht University, Universiteitssingel 40, 6229 ER Maastricht, The Netherlands

[5] Institute for Clinical Diabetology, German Diabetes Centre, Leibniz Centre for Diabetes Research at Heinrich Heine University, Auf'm Hennekamp 65, Düsseldorf, Germany

[6] These authors contributed equally

*Corresponding Authors: Mahmoud H. Elbatreek, Karin Jandeleit-Dahm and Harald H.H.W. Schmidt,

Emails: melbatreek@ppmlab.net, karin.jandeleit-dahm@monash.edu, hschmidt@ppmlab.net

Supplementary Figures and Legends

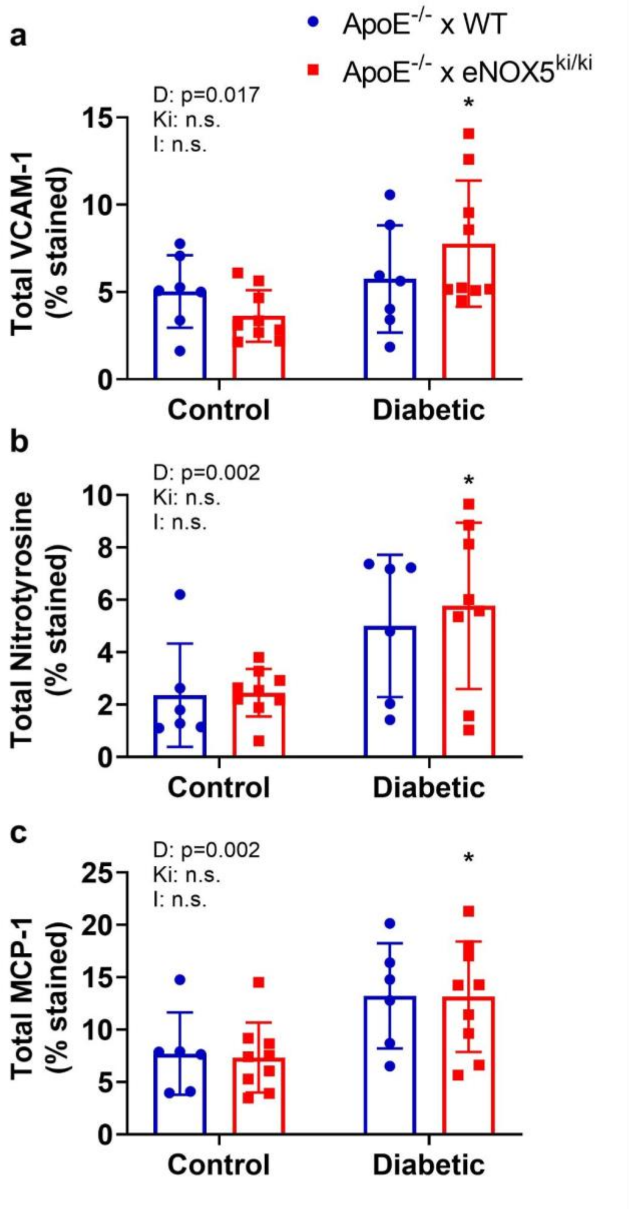


**Supplementary Fig. S1.** Analysis of immunohistochemical staining for various antibodies in aorta from ApoE $^{-/-}$ mice (ki/ki) and without (WT) eNOX5 expression. Representative images are shown in Supplementary Fig. S2. Negative control slides are shown in Supplementary Fig. S4. ND: not diabetic; Diab.: diabetic.  2-way ANOVA: D- relative to diabetic status; Ki- relative to genotype (Nox5); I- interaction. Tukey post hoc t-test p<0.05 * vs. relevant non-diabetic control.

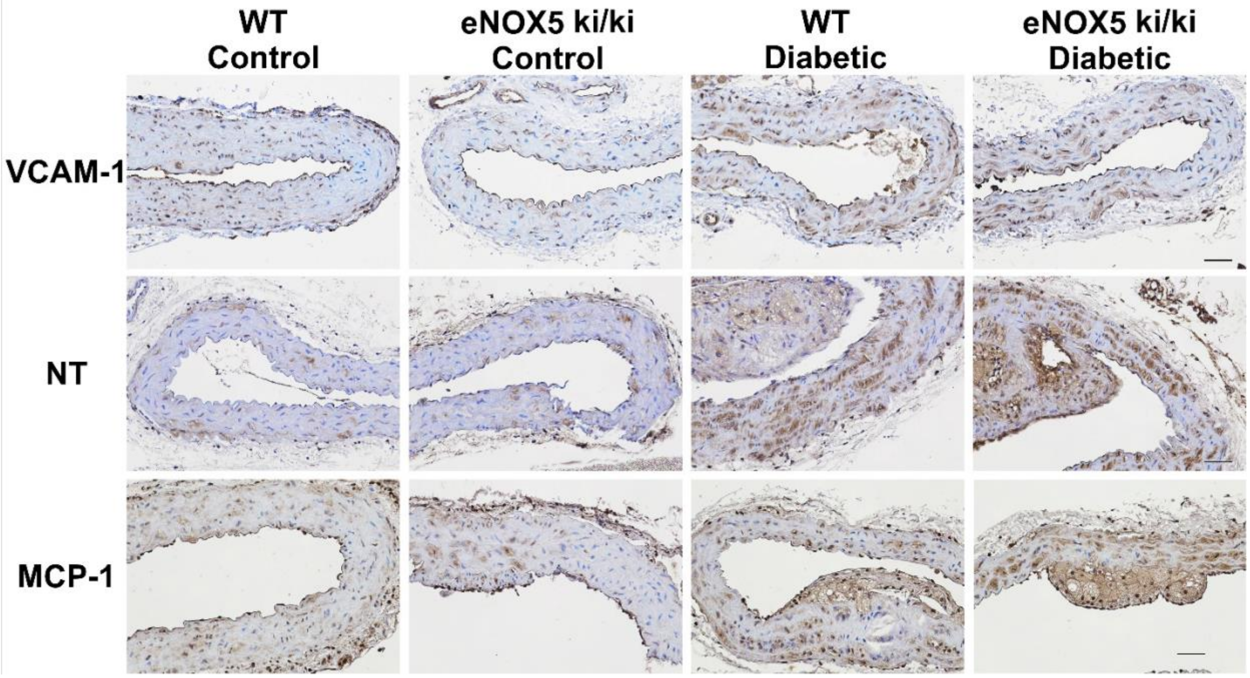


**Supplementary Fig. S2.** Representative images of immunohistochemical stains analyzed in Fig S1. from ApoE $^{-/-}$ mice with (ki/ki) and without (WT) eNOX5 expression. Scale bar represents 50µm. Negative control slides are shown in Supplementary Fig. S4.

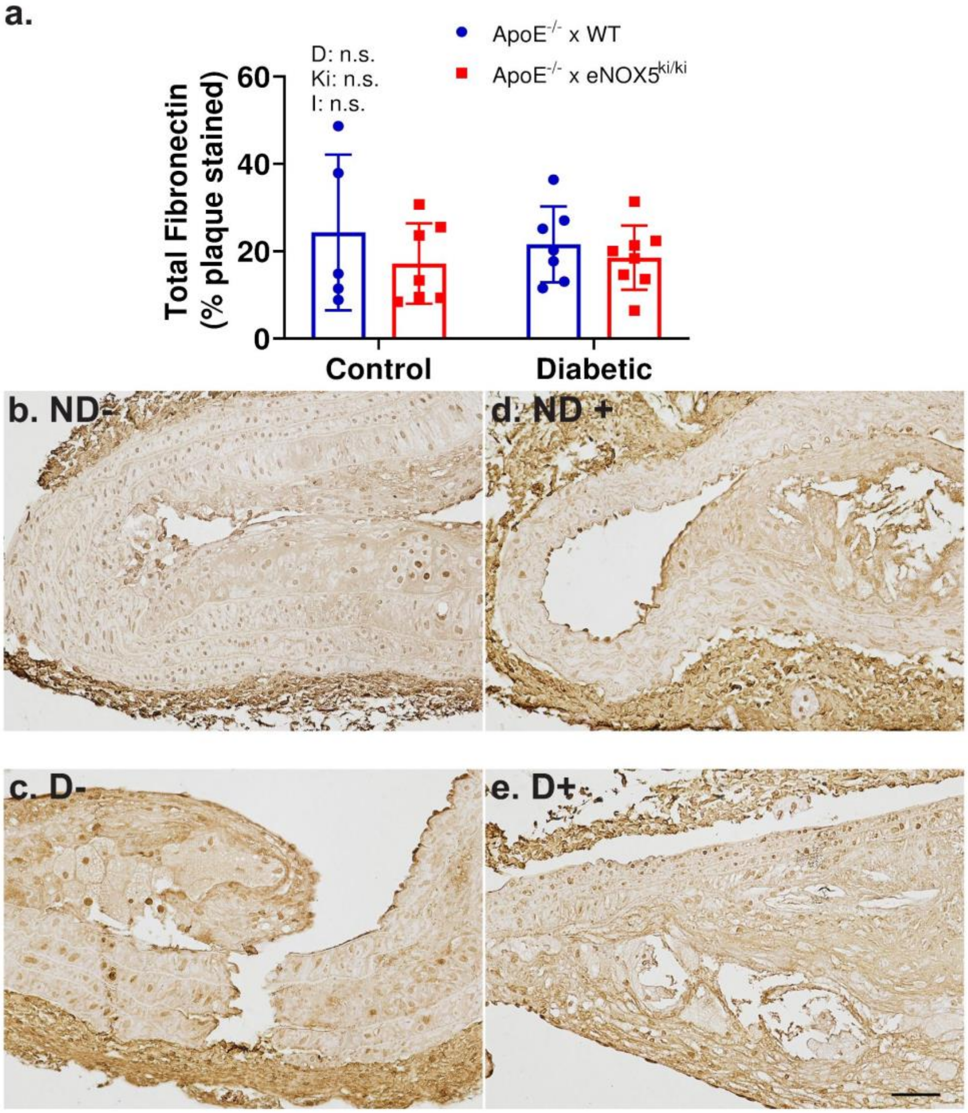


**Supplementary Fig. S3.** Assessment (a) of fibronectin accumulation in atherosclerotic plaques and adjacent wall (Dark brown DAB staining) from aorta in ApoE $^{-/-}$ mice without eNOX5 (b, c) and with eNOX5 expression (d, e) in non-diabetic (ND; b, d) and diabetic (c, e) animals. Note that ND animals have much less atherosclerotic plaque than diabetic mice. 50µm scale bar. 2-way ANOVA: D- relative to diabetic status; Ki- relative to genotype (Nox5); I- interaction. Negative control slides are shown in Supplementary Fig. S4.

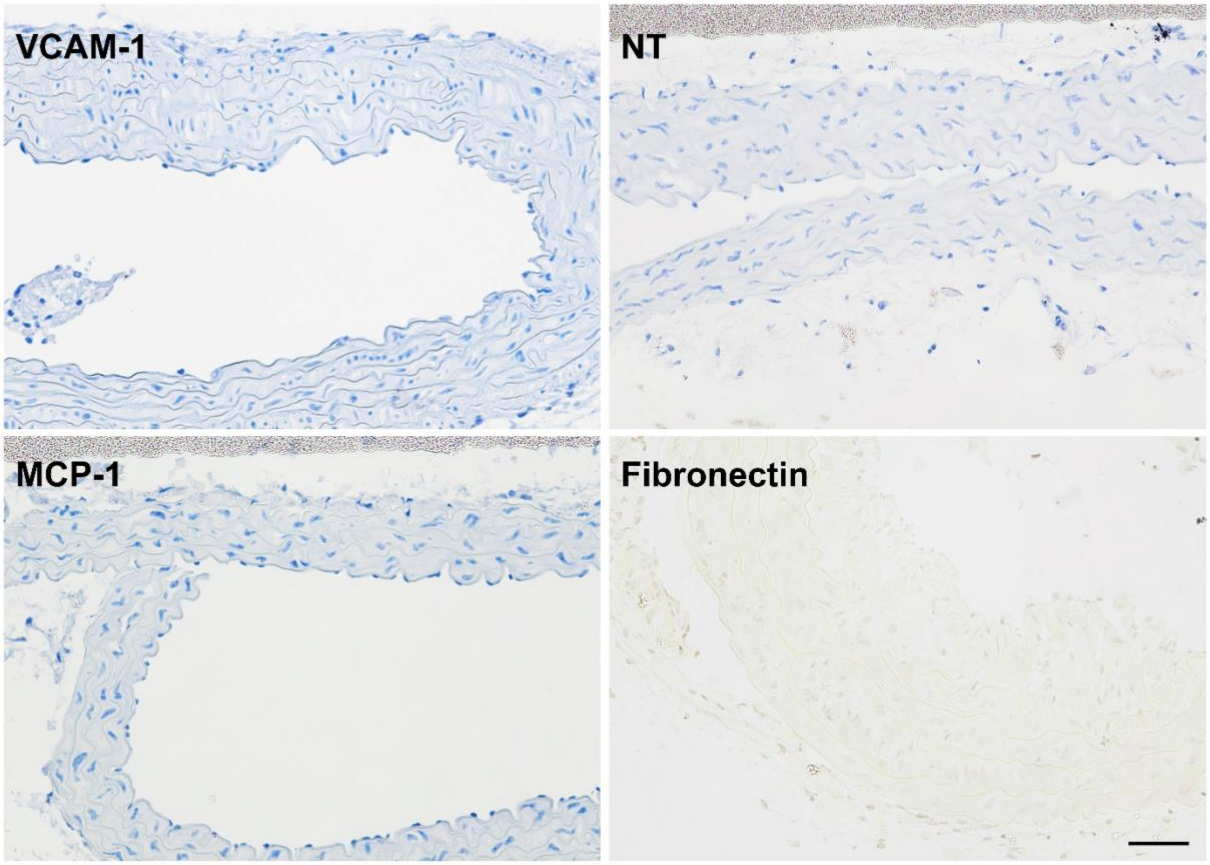


**Supplementary Fig. S4.** Negative control slides (where the primary antibody was omitted) run with immunohistochemistry presented in Fig s1-3. Note there is no hematoxylin counterstaining for the fibronectin negative control. Scale bar represents 50µm.

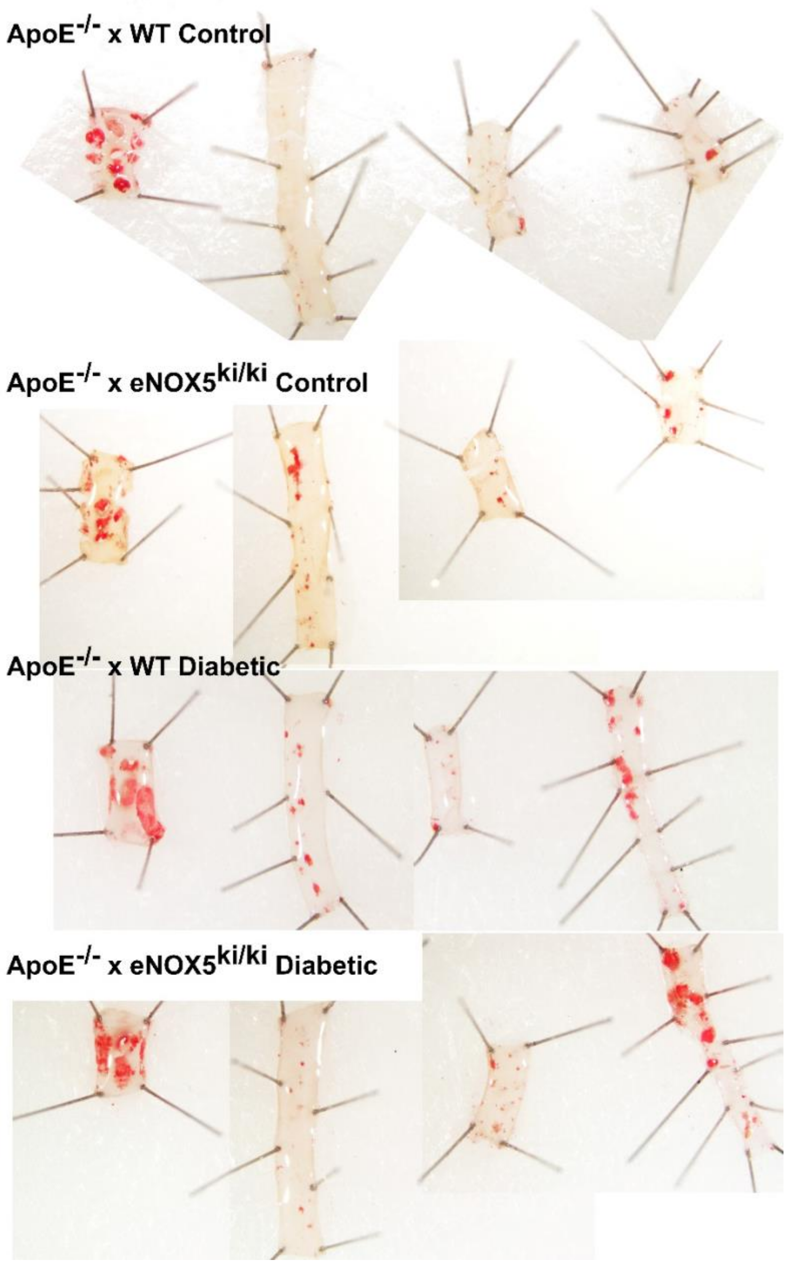


**Supplementary Fig. S5.** Representative images of *en face* mouse aorta from ApoE $^{-/-}$ mice with (ki/ki) and without (WT) eNOX5 expression stained with sudan IV for quantification of intimal plaque area assessment presented in Figure 2. Aortic sections (left -right): arch, thoracic, abdominal (to renal arteries), abdominal (to iliac bifurcation)

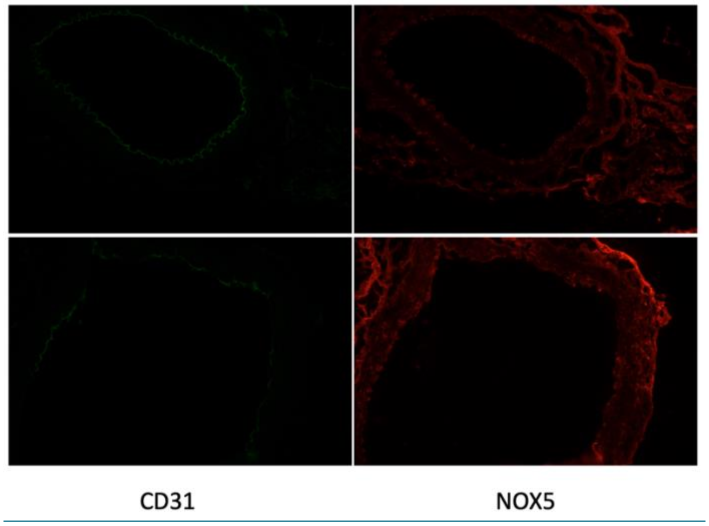


**Supplementary Fig. S6.** Representative images of immunofluorescence staining of NOX5 and CD31 showing expression of NOX5 in endothelial cells of eNOX5$^{Ki/Ki}$ mice

**Supplemental Table 1.** RT-PCR probe and primer sequences.

| **Mouse genes of interest** | | **Nucleotide sequence** |
|---|---|---|
| MCP1 | Probe sequence | 6- FAM AATGGGTCCAGACATAC |
| *Monocyte chemoattractant protein-1* | Forward primer 5'-3' | GTCTGTGCTGACCCCAAGAAG |
| | Reverse primer 3'-5' | TGGTTCCGATCCAGGTTTTTA |
| VCAM1 | Probe sequence | 6-FAM CCAAAATCCTGTGGAGCAG |
| *ascular cell adhesion molecule-1* | Forward primer 5'-3' | CTGCTCAAGTGATGGGATACCA |
| | Reverse primer 3'-5' | ATCGTCCCTTTTTGTAGACATGAAG |
| F4/80 | Probe sequence | SYBER |
| | Forward primer 5'-3' | GGTACAGTCATCTCCCTGGTATGTCT |
| | Reverse primer 3'-5' | GGTTCTGAACAGCACGACACA |
| NFkB | Probe sequence | 6-FAM AGCTCAAGATCTGCCG |
| *Transcription factor p65, RelA* | Forward primer 5'-3' | TCTCACATCCGATTTTTGATAACC |
| | Reverse primer 3'-5' | CGAGGCAGCTCCCAGAGTT |
| NOX2 (gp91phox) | Probe sequence | 6- FAM CAACTGGACAGGAACCT |
| | Forward primer 5'-3' | AGTGCGTGTTGCTCGACAAG |

| | | |
|---|---|---|
| *NADPH oxidase beta subunit gp91phox* | Reverse primer 3'-5' | CCAAGCTACCATCTTATGGAAAGTG |
| NOX4 *NADPH oxidase 4* | Probe sequence | 6- FAM CATTTTGCTATTTCATCAAA |
| | Forward primer 5'-3' | AAAATATCACACACTGAATTCGAGAC T |
| | Reverse primer 3'-5' | TGGGTCCACAGCAGAAAACTC |
| HO1 *Heme oxygenase 1* | Probe sequence | 6- FAM CTAAGACCGCCTTCCT |
| | Forward primer 5'-3' | AGATGACACCTGAGGTCAAGCA |
| | Reverse primer 3'-5' | TTGTGTTCCTCTGTCAGCATCAC |
| GPx1 *Glutathione peroxidase 1* | Probe sequence | 6- FAM CGACCCCAAGTACATC |
| | Forward primer 5'-3' | CCCCACTGCGCTCATGA |
| | Reverse primer 3'-5' | GGCACACCGGAGACCAAA |
| NRF2 *Nuclear factor erythroid 2 like 2* | Probe sequence | 6- FAM CACAGTGCTCCTATGC- |
| | Forward primer 5'-3' | AGCCTCTGTCACCAGCTCAAG |
| | Reverse primer 3'-5' | TGTTGTATTTTCACATTGGGATTCA |
| Collagen IV *Procollagen type 4* | Probe sequence | 6- FAM CAGTGCCCTAACGGT |
| | Forward primer 5'-3' | GGCGGTACACAGTCAGACCAT |
| | Reverse primer 3'-5' | GGAATAGCCGATCCACAGTGA |

| | | |
|---|---|---|
| Fibronectin | Probe sequence | 6- FAM CCCCGTCAGGCTTA |
| | Forward primer 5'-3' | ACATGGCTTTAGGCGGACAA |
| | Reverse primer 3'-5' | ACATTCGGCAGGTATGGTCTTG |
| CTGF<br>*Connective tissue growth factor* | Probe sequence | 6- FAM ACTGCCTGGTCCAGAC |
| | Forward primer 5'-3' | GCTGCCTACCGACTGGAAGA |
| | Reverse primer 3'-5' | CTTAGAACAGGCGCTCCACTCT |
| MMP2<br>*Matrix metalloproteinase 2* | Probe sequence | 6- FAM TGCACCAGCGCCGG |
| | Forward primer 5'-3' | TCACTTTCCTGGGCAACAAGT |
| | Reverse primer 3'-5' | GCCACGAGGAATAGGCTATATCC |
| MMP9<br>*Matrix metalloproteinase 9* | Probe sequence | 6- FAM CATCAAAAACATCCACATTG |
| | Forward primer 5'-3' | TGAGTCCGGCAGACAATCCT |
| | Reverse primer 3'-5' | CGCCCTGGATCTCAGCAATA |
| PKCα<br>*Protein Kinase C - alpha* | Probe sequence | 6- FAM CGATCCCAGTCCCAG |
| | Forward primer 5'-3' | AGACAAAGACCGGCGACTGT |
| | Reverse primer 3'-5' | TTAGCTCTGAGACACCAAAGGAAA |
| PDGF | Probe sequence | 6- FAM TCGCGGAACCTC |
| | Forward primer 5'-3' | TGTAATCGCCGAGTGCAAGA |

| | | |
|---|---|---|
| *Platelet derived growth factor* | Reverse primer 3'-5' | CATTGCACATTGCGGTTATTG |
| CD36 | Probe sequence | 6- FAM AGAATCTGAAGAGACCTTAC |
| | Forward primer 5'-3' | AAGCCAGCTAGAAAAATAGAAGCATT |
| | Reverse primer 3'-5' | GTCTCATTTAGCCACAGTATAGGTACA A |
| ACAT1 | Probe sequence | 6- FAM CTGCCGTAGACCCCAT |
| *Acetyl-coenzyme A acetyltransferase 1* | Forward primer 5'-3' | TGGCACGAATTGCAGCAT |
| | Reverse primer 3'-5' | GCAGGCGCAAGTGGAAAAT |
| ABCA1 | Probe sequence | 6- FAM ACTCCACATAGAAGACTACT |
| *TP-binding cassette 1, subfamily A, member 1* | Forward primer 5'-3' | TCCTCTCCCAGAGCAAAAAGC |
| | Reverse primer 3'-5' | TGGTCAAGTGTTGTCTGAGAGAGA |
